# How do staff work in NHS hospital operations management meetings to support resilience in everyday service delivery? A qualitative study

**DOI:** 10.1186/s12913-025-12229-3

**Published:** 2025-01-21

**Authors:** L. Sutton, C. Tarrant, J. Willars, T. Coats, M. Simmonds, D. Mclean, A. Boyle, K. Dreesbeimdiek, S. Richter, A. Oyedijo, D. Roland

**Affiliations:** 1 SAPPHIRE Group, Population Health Sciences, Leicester University, Leicester, UK; 2 NIHR Greater Manchester Patient Safety Research Collaboration (GM PSRC), National Institute for Health and Care Research, Manchester, UK; 3 Emergency Medicine Academic Group, Cardiovascular Sciences, Leicester University, Leicester, UK; 4 Nottingham University Hospitals NHS Trust, Nottingham, UK; 5 Cambridge University Hospitals NHS Foundation Trust, Cambridge, UK; 6 Department of Medicine, Cambridge University, Cambridge, UK; 7 Department of Management, Hankamer School of Business, Baylor University, Waco, TX USA; 8 Paediatric Emergency Medicine Leicester Academic (PEMLA) Group, Children’s Emergency Department, Leicester Royal Infirmary, Leicester, LE1 5WW UK

**Keywords:** Management, Operations Management, Healthcare System, Hospitals

## Abstract

**Background:**

Operations Management meetings in NHS hospitals provide an opportunity for operational and clinical staff to monitor demand and capacity and manage patient flow. These meetings play an important role in the achievement of resilient performance over time. However, little is known about the work that takes place within these meetings in the United Kingdom’s National Health Service.

**Methods:**

We conducted a qualitative study observing 29 Operations Management meetings across three English hospitals between June and October 2023. The observations focused on: who was present; how meetings were organised and conducted; what data were used; how decision-making took place; and the types of work that were undertaken. We also conducted 17 semi-structured interviews with divisional leads and meeting chairs. A grounded theory analytic approach involved exploring the data in two sites to identify key themes, and then testing these themes through a third comparator site.

**Results:**

We identified the type and extent of work that took place in these meetings to maintain flow and enable resilient service delivery. Operations Management meetings provided an opportunity for staff to come together to engage in collective sense-making, to develop a shared mental model of the state of the hospital and to build a collective understanding of where action was needed. Review of centralised data, formally encoded and recorded in numerical form, played an important role, but staff also drew on local intelligence to make sense of and adapt to often pressurised situations. We identified three types of work: Sense-making and Interpretation, and Risk work (which together contributed to maintaining organisational function) and Maintaining morale (which supported individual staff resilience).

**Conclusions:**

The work that went on in Operations Management meetings functioned to support organisational and individual resilience, through staff repeatedly sharing and assessing information on capacity and demand, taking action to address these continually changing pressures, and having their efforts recognised.

**Supplementary Information:**

The online version contains supplementary material available at 10.1186/s12913-025-12229-3.

## Background

In all hospitals where acutely unwell patients are admitted there are usually ‘Operations Management Meetings’, which are held online or face to face up to four times a day throughout the week. These have a variety of names such as ‘bed meetings’, ‘flow and capacity meetings’, ‘command centre meetings’ etc. and are for managers and clinicians to discuss patient flow through the hospital and determine strategies to optimise bed availability and patient discharge. Bed availability is a priority for hospital management as hospitals routinely run at more than ideal capacity. The King’s Fund suggest that hospitals with more than 85% bed occupancy can expect to have regular bed shortages, periodic bed crises, cancellation of elective, unused operation capacity and increased numbers of health care-acquired infections [1]. National Health Service (NHS) hospitals in the United Kingdom routinely run on > 90% bed occupancy, resulting in increased mortality [2] and poor patient experience.

Operations Management Meetings (henceforth Operations meetings) utilise data collected from areas in which patients are admitted (such as the Emergency Department (ED) and Assessment unit) to determine the amount of inflow, combine with information about hospital wide discharges (outflow) to manage occupancy (inflow – outflow). Information on factors such as staffing, sickness levels, and external events impacting on capacity and demand is taken into account in meetings to inform planning about the management of bed occupancy and flow over the next few hours. Meetings are chaired by a manager with responsibility for hospital flow, and involve representatives from across a hospital’s directorates and divisions, who attend to present information about their area and to discuss any actions needed.

Operations meetings, with their remit to review and manage hospital capacity and demand over short windows of time, act as points at which ‘on-the-day’ adaptations [3] and adjustments in service organisation and delivery can be identified and agreed, arguably contributing to the achievement of resilience within NHS Trusts. The concept of resilience relates to the ability of organisations and systems to cope effectively with unexpected incidents or threats, recover from crises, and build success for the future [4], enabling them to sustain everyday performance when faced with expected challenges as well as unanticipated conditions [5]. The Concepts for Applying Resilience (CARE) model [6] describes resilience as involving adaptations and adjustments in everyday work, and Anderson et al. highlight the importance of adaptive capacity for resilience—the ability of healthcare organisations and systems to make adaptations and adjustments in the face of fluctuating demand and capacity, in order to deliver consistently high quality and safe care [6]. Anderson et al. argue for the need to shift from a focus on the resilience in relation to the delivery of clinical care by health professionals, towards a focus on the role of managers, hospital boards, and other actors who play a key role in organisational resilience away from frontline care delivery [7]. Much attention has been paid to heath system resilience in the face of disasters or severe shocks such as the Covid-19 pandemic [8]. We suggest a need for more attention to resilience in the context of chronic pressures, such as the high levels of demand faced in hospital acute care: the ‘winter pressures’ that continue to be felt all year [9]. In this study we aimed to explore the work that was undertaken by staff in operations management meetings to support resilience in service delivery on a day-to-day basis.

## Methods

We conducted a qualitative study involving observations of Operations meetings, and semi-structured interviews with divisional leads and chairs of operations meetings, in three NHS teaching hospitals in different regions of England. Organisation A is a city-based Trust that has 1,991 inpatient beds. It serves a large and a diverse population and has three hospital sites. Organisation B is a large city-based Trust. It has around 1,700 in-patient beds with two hospital sites. Organisation C is also a large city-based teaching healthcare provider with approximately 1,200 in-patient beds in two hospital sites. Observations and Interviews initially occurred in organisations A and B where initial themes were derived and then tested and confirmed with further observations in organisation C. Observations and interviews were initially directed at understanding how senior clinical and managerial staff used data in their decision making at operational meetings, but based on initial observations and interviews, we broadened our focus to exploring how information (data about capacity and resources, and soft intelligence) was used in operational management meetings.

Two experienced qualitative researchers observed a total of 29 meetings at different time points and on different days of the week between June and October 2023. Our research was facilitated by senior NHS managers who provided access to meetings and suggested key staff to approach for interview. Two sites, Organisation A and Organisation B conducted meetings online, and face-to-face meetings took place in Organisation C. A single researcher attended each meeting to observe virtually or in-person as appropriate. The observations focused on who was present, how meetings were organised and conducted, what data were used and how decision-making took place. Observations were documented in field notes using an observation guide and structured data summaries produced for each meeting observation. Audio-recorded field notes were also made after each meeting observation.

Semi-structured interviews with divisional leads, managers, and meeting chairs took place via Microsoft Teams or by telephone, and were audio-recorded with the participants’ agreement. Interviews lasted between 40 to 60 min. A topic guide was developed by CT and LS which explored participants roles, use of data and views and experiences of taking part in the meetings (see Appendix 1). Participants were recruited through the site lead who e-mailed divisional leads and key personnel introducing the researchers and asking them to contact the research team should they be interested in participating in the study. Audio-recorded field notes and Interview recordings were transcribed verbatim and anonymised, and transcripts were uploaded into Nvivo 12 qualitative data software for data management.

We drew on a modified grounded theory approach [10], collecting and analysing data iteratively. “Through our analysis we identified connections with resilience theory, which helped shape our interpretation of findings.”

LS and CT individually read through several transcripts and data summaries, and open coded them to draw together a coding frame with which to code the remaining data. Key themes identified related to the way that the meetings were orientated, the types of information shared, the process of action planning and decision-making, and the expression of praise and support in meetings. As analysis progressed, we used narrative summaries to synthesise and interpret the data. Initial findings were discussed with the wider project team, including clinicians and managers with expertise in Operations Management, who contributed to data interpretation.

## Findings

### Operations meeting format

Operations meetings took place up to four times a day, lasting between 15 and 30 min and involving between 10 and 15 directorate leads, estates and facilities managers and flow matrons/managers. These meetings were informed by, and followed up through, other meetings at a directorate level that took place throughout the day. Operations meetings were chaired by senior operations managers, who co-ordinated the information shared and were responsible for action-planning. In every site, they had a digital central bed-state data system available to them, which displayed near-real time data on the organisation’s capacity (bed numbers and identified discharges, or queries for discharge) and demand (e.g. the number of patients on route to the ED, in ED and waiting times). The exact specification of these systems and types of information available varied between trusts. All attendees in Organisation A and Organisation B had access to the centralised data display, which they were able to view on their own computer screens if they chose to do so. The bed state system in Organisation B had been expanded to also include easily accessible information about predicted discharges and potential delays to discharge. In Organisation C, meetings were held in person in a pre-booked room, containing a large screen where the data display was visible for everyone attending. During meetings, a representative from each division or clinical group (such as the Emergency Department, Cancer specialities, Surgery) was responsible for providing an update on their bed state including the numbers of beds they had available in their speciality or department.

### Primary activities

Our observations and interviews suggest three primary activities undertaken in these frequently repeated meetings: Sense-making and Interpretation; Risk work; and Maintaining morale. Detailed supportive quotes and analysis can be found in Appendix 2; the below is an overview of the core themes (Table 1).

**Table 1:** Summary of the three primary activities undertaken in operations meetings

Theme Heading	Sub-Heading	Summary
Sense-making and Interpretation	Review of Bed Data	Core information on beds available, beds needed and current inflow and outflow numbers
	Accessing Soft Intelligence	Knowledge about potential enablers and barriers not available through routinely collected data
	Anticipating Future States	Utilising known events and data to predict capacity challenges
	Discharge Predictions	Understanding how flow may be affected by planned discharges
	Building a collective understanding	Collective interpretation of information about demand and capacity
Risk Work	Identifying Risks	Using data and experience to understand the risk posed by the current and future state of the hospital
	Prioritising Risks	Identifying the key areas of capacity challenge that need to be addressed
	Allocating Responsibility	Identifying who is tasked with delivering the required change or intervention
Maintaining morale	Recognising Success and Displaying Empathy	Understanding and mitigating the emotional challenge of working in a system which is consistently over-capacity

### Sense-making and interpretation

A key aspect of work within Operations meetings involved enable operations managers and divisional leads engaging in sense-making and interpretation, to construct a shared understanding of the current state of the trust. Sense-making and interpretation of data and intelligence took effort and skill.

#### Review of bed data

Centralised data held on bed-state data systems included bed state numbers, number of critical care beds, step down beds, numbers of patients waiting, number of ambulance calls, number of infections, number of beds closed and staffing numbers. Key elements of this information were shared by the chair of the meeting to provide a high-level overview of the state of the organisation.

Divisional staff reports during the meeting provided real time updates to the formally recorded data, summarising their bed capacity and highlighting any issues.

*Specialty Directorate giving status “9 with 2 later. Assessment Unit at (name of hospital) have 1 now, 3 later.” [Meeting participants] talked about ward x to look to transfer if busy. “Neonates at Opel 3* = *37 babies at [name of hospital]. Obstetrics – acuity 65” (Organisation A , OBS 03).*

Having a clear awareness of the overall state of the organisation was supported by review of the data held on the central system, but also involved a shared and distributed understanding of the unfolding situation on a daily basis, and the local conditions that could impact on this. Bed state information had to be contextualised through sharing and taking into account local intelligences from across the divisions.

#### Accessing soft intelligence

Locally-held soft intelligence included: knowledge about problems or delays arising from estates and facilities issues such as lift breakdowns or cleaning requirements; knowledge about patients who might be challenging, or require additional support or staffing resource, and/or equipment such as specialist beds and hoists; staffing changes and sickness; and informal knowledge and experience of their division and site.

Chairs described the importance of accessing soft intelligence from divisional leads about the state of their bed capacity and staffing.

*Your soft intelligence is the bit when you’re actually having that conversation with people, they’ll tell you, (the reporting service) It looks like that but actually it’s not that, or, you can’t see the staffing issues from that screen, it doesn’t tell you, so you have to use all the forms of communication with them to actually ask if we’ve got any staffing issues and can we use those* (Organisation B, INT05).

#### Anticipating future states

Staff participating in Operations meetings had to consider events or conditions that could place additional demand on the service over the next few hours such as activity in other healthcare settings (i.e. need for potential ambulance transfers from other hospitals), local public events and unique events (e.g. industrial action) and their implications.

*Silver lead did make a prediction here that as at the organisation there was a festival on that it could get busy and more imaging would be required. [Divisional lead] said that she was very aware of this prospect and was anticipating a busier afternoon / evening.* (Organisation A, OBS 01)

Chairs in Organisation A in particular, described the critical role of informal, intuitive knowledge and experience in enabling them to work out, in collaboration with divisional leads, where pressure points were likely to be over the next few hours. This type of knowledge remained unquantifiable and unmeasurable.

*But you know when you’ve been doing it for a whileyou have a background in your head of what..soSurgery might say they’re minus 23, but their figures will assume that they’ve got 23 people in triage, and actually experience tells you that at least 50 percent of those people will go home. So you look at that and think well that might be OK. I mean in theory you could have one of those days where every single patient comes in, but generally they don’t. So you’re thinking if they’ve got 10 beds and 20 people in triage, they’re probably gonna get to a zero, and you sort of balance the risk off in your head. But experience tells you that. (Organisation A , INT01)*

#### Discharge planning

Those in the meeting needed to consider planning for discharges within their wards and divisions. In Organisation B, predictive information on potential discharges was available on their centralised systems as a discharge profile, whereas in Organisation A and Organisation C discharges tended to be reported by representatives from each division.

*[Operations lead] OPEL 4 in ED, Surgery and critical care – others on RED. Longest waiting patient 41 hours for a gastro bed. 57 patients waiting. Discharge profile dire with 91 queries.. We need a clear plan on discharge otherwise worse tomorrow. (Organisation B, OBS 4)*

*[Person 5] from one of the hospitals gave the report. Complex discharges – 2 discharged, 2 waiting allocation, 1 being made ready to travel by ambulance, 1 TTO to be written on transport. (Organisation A, OBS 1)*

- OPEL (Escalation Level)
- ED (Emergency Department)
- TTO (To Take Out; medications a patient is discharged with)

Predicting discharges could be at an individual level (the treating team think the patient can go home tomorrow) or at a system level (at a specific time of year there tend to be similar amounts of discharges). There was some doubt about the extent to which predictions could be made accurately. Such data was specific, partial and constructed and required more work to interpret.

While patients may be identified as being suitable for discharge, there could be problems getting care packages into place or a patient may experience a sudden worsening of their condition. Meaning that:

*A bed is not empty…Until there’s not a patient in it* (Organisation C, INT 005).

#### Building a collective understanding

Data and intelligence shared had to be interpreted to construct meaning and identify implications. Chairs had to make sense of the information to understand the implications for capacity and flow across the organisation, in order to identify areas of risk and prioritise actions. Divisional representatives had to listen, translate and make sense of each piece of information shared and undertake work to understand what it meant to their division and how it might impact on other parts of the organisation. Chairs and divisional leads discussed how data from one division impacted on demand and requirements from other divisions in order to manage flow. Participants had to re-assess their own division’s capacity following the breakdown of the types of patients they were receiving, who they could move from where and whether they could take additional patients on specific wards.

*The chair asked about* <*organisation 3*> *and the numbers of beds were shared. ] The chair commented that this was really good capacity, however, there are a lot of patients in A&E with no plans and there are 30 patients going on the bed list in a few hours and that will “be your [full] capacity and more”*. (Organisation A, OBS09).

### Risk work

Risk work in this context describes the human effort, in combination with the material infrastructure of data systems and Operations meeting routines, that come together to understand and organise risks [11]. Risks were considered in the context of the collective understanding of the current state of the organisation in terms of demand and capacity.

#### Identifying risks

Areas of risk could be revealed through the sharing and interpretation of information about bed states, capacity, anticipated future states, and local conditions. They could also be brought to the fore through deliberate highlighting of known risk areas by the chair or divisional representatives.

*ED representative [from the organisation] wanted to flag that ED was challenged from a nursing perspective. Seven gaps which means that they can only take four ambulance handovers. That may be increased to eight but there is a risk to ambulance handovers (Organisation B, 03A)*.

*The hospital was under pressure, many people were waiting to be seen which was highlighted as a huge risk for them (Organisation C, OBS02A).*

#### Prioritising risks

After identifying potential risks, the chair set the priorities in discussion with participants articulating what staff needed to focus on to improve the situation, or ensure that it did not deteriorate further. This informed the chairs review and discussion of the options available to them, action planning and task allocation.

*Close ED corridor by midday at the latest. Involves reducing the wait for [ward] – hopefully by the next meeting. Delayed step downs from the 29th need to be placed by the 1pm meeting. Need to fill all available capacity within division. (Organisation B, 03A)*

#### Allocating responsibility

After highlighting areas to prioritise, the chair and participants at the meeting aired and discussed potential options to mitigate identified risks. Divisional leads reported particular issues or problems their division was facing, usually involving staff shortages, complex patients or IT or equipment failures. Based on collective understanding obtained in the meeting, staff identified solution options to the respective situations which were often limited but they were aired and negotiated. These focused, for example, on whether to close or open a particular area, or move patients from one place to another.

*Discussion about if we need to close the discharge lounge can we use the nurses better elsewhere? Discussion about night staff. Chair said “will look to see what’s happening in the discharge lounge later, carry on for now. If we are to close discharge lounge will then relocate nurses, let me make some phone calls and will let you know”. (Organisation A , OBS 03)*

Divisional leads reported when they had no capacity, or had staff shortages then all options needed to be considered to resolve the situation. In the following extract the chair identified the option of using the Cancer division’s spare capacity for other patients.

*Cancer were at amber. Have capacity. Need to place surgical and medical electives. No capacity on oncology. Bed manager is working on getting an oncology bed. Lead – great something about using capacity for outlying respiratory patients or those on HCOP but to check with bronze. (Organisation B, OBS07).*

However, there were instances where the options aired were not viable. In the following extract other options were not discussed following a request for others to help transport patients.

*Chair asked if a specific team could “crew up” to help. ED representative who was reporting said that she thought this might be possible but also gave some reasons why this might not work. She said that the problem is that they support another area now which means that they are very limited on what they can do. (Organisation A , OBS 01)*

Action-planning and goal setting (including following up problems) was primarily undertaken by the chair who summed up actions at the end of the meeting by asking staff to focus on key areas and prioritise certain issues.

*Chair summed up with their priorities which were to support E12, start theatres, work through B3 list and look at everything on discharge – 80 queries so push on transfer of care and discharge and get D2As submitted*. (Organisation B OBS05)

### Maintaining morale

#### Recognising success and displaying empathy

The work of the operations management team involved having to manage a high level of demand with limited capacity on an ongoing, day to day basis. They had to manage and balance risks, negotiate and make difficult decisions, constantly revisiting the situation with frequently shifting resources. The interviewees reported that they faced chronic stressors every day and that each day felt the same as the last. It could be draining and demoralising for all involved.

*The meeting is a bit Groundhog Day because you know, there’s long waits on ED, There’s a lot of patients waiting for medicine in ED, so it will be the same.] I’m not exaggerating much by saying every day you’ll hear the same problem, so that can be quite tiring or jading is the right word…We're always, always under pressure.* (Organisation A INT01A).

Very often, this meant that participants were wary and anxious about what they were about to face when they accessed the data before the meeting.

*You just think oh my goodness, what am I coming into? …You just have that heartsink as soon as you turn it on, going what am I coming into? (INT 04, Organisation B)*

In many meetings we observed work that primarily functioned to support and reinforce individual resilience in senior managers or clinicians present. Chairs worked to ensure a sense of community and belonging, with a sense of collective working towards common goals. Given the sense of futility that could be attendant to Operations meetings, work to establish clarity of purpose and achievable goals played an important role. In some cases chairs worked to keep the focus on issues they could all address, rather than those that they could not. With the emphasis on targeted discharges on a daily basis at Organisation B, it became possible to observe and measure this particular aspect of success.

*Lead summed up with their priorities which were to support [department], start theatres, work through [list] and look at everything on discharge – 80 queries [possible discharges] so push on transfer of care and discharge. (Organisation B OBS05)*

Chairs also emphasised the maintenance of momentum in addressing patient flow as a goal in itself, even if the absolute overall position of the trust in terms of capacity status changed little from meeting to meeting.

We observed chairs working to maintain morale through encouragement, praise and gratitude.

*First mentioned was the success in getting the corridor shut, she thanked everyone for their support and that ticking that box meant it was a better experience for the patient even if it made ED busy.* (Organisation B, OBS02B)

*Great team work in getting patients in right place. Keep going*. (*Organisation A , OBS 05*)

Displays of coping from divisional leads also seemed important for collective morale. The participants let the chair and others know how their division was doing, whether they were coping with demand or whether they needed support. When faced with problems to solve, divisional leads often responded by stating that “we will do our best” or that they could cope. We observed work undertaken by participants to demonstrate that their own division was up to the task, and that they could cope with the pressure.

*Working through outlier capacity but nothing to worry about at the moment. Said that they will be fine and “will hold their own this afternoon” (Organisation B, OBS 01B)*

## Discussion

Our findings provide an insight into work undertaken by staff in Operations meetings that supported day-to-day resilience to routine pressures in NHS Trusts. Drawing on the Concepts for Applying Resilience (CARE) model of resilience [6], we can see Operations meetings as spaces in which resilience work is done; in which alignments between demands and capacity are repeatedly reviewed and assessed, and options for adaptive ‘on-the-day’ adjustments [3] identified, discussed, and agreed. Considering McCrae’s Moments of Resilience [12], these meetings operate at a micro level to enable situated resilience. Our findings describe the work that is involved in supporting this situated resilience within Operations meetings, which involves bringing together formally encoded data with local intelligence, engaging in collective sense-making and interpretation, and risk work. By understanding Operations meetings as Moments of Resilience, our research highlights the importance of considering situated resilience beyond the localised management of unexpected events at the frontline of care delivery, to encompass the every-day work that is done within NHS trusts to constantly monitor and adjust to fluctuating capacity and chronically high levels of demand that persist over time.

Our findings highlight for the importance of collective sensemaking about the current status of capacity across the healthcare organisation and the demands or threats that the organisation is currently facing. The staff we observed were clearly highly skilled in performing resilience in Operations meetings. They continually turned up, made do with the difficult circumstances they found themselves in, provided support to each other and tried to energise staff into finding solutions.

Thinking “managerially” is one way that senior staff who are specifically concerned with capacity and demand within the context of fluctuating resources in a wide range of settings are able to enact resilience. In healthcare it has been shown to occur when making decisions about the place of care for babies born pre-term [13] and in multi-disciplinary team meetings for older people with long-term conditions [14]. The latter study found similar sense-making actions occurring within these meetings to our findings. These included; information sharing and seeking, solution seeking and decision making and task allocation. This work, in line with ours, points to the way that these behaviours contribute to resilience in situ in different healthcare contexts. More broadly, our findings align with key elements of leadership behaviours that have been identified as promoting adaptive capacity in hospital teams [15].

Our findings also highlight the hidden work that happens within Operations meetings to maintain and support staff morale in the face of chronic pressures. Operations meetings provide an opportunity for monitoring and bolstering the individual resilience of staff who have responsibility for maintaining flow across the hospital: a task that for many can seem to be an endless challenge. This work is done by the managers in charge of the meetings; providing more evidence of the important role that managers are argued to play in supporting staff resilience [16]. This work is also done collectively through meeting participants acknowledging the difficulties they face and providing mutual support and encouragement. In line with research by Agostini et and colleagues [17] these meetings highlight the importance of social and behavioural aspects and social interactions at play in fostering organisational and individual resilience. Our findings highlight the intertwining of work to achieve situated resilience, and to support individual resilience, that happens collectively within Operations meetings. An immediate implication of our finding is for system leaders to be aware of the importance of these meetings in relation to resilience and not just performance metrics and provide suitable resource and training for this.

Previous research has advocated for the enhancement of operations management knowledge while considering future theoretical advancements within the discipline [11]. Our research has implications for the Resource-Based View (RBV) theory [18, 19]. The RBV posits that an organisation’s resources and capabilities can enhance performance. This study has demonstrated that the work done within Operations meetings enables identification and establishing of consensus on on potential alterations in service organisation and delivery, thereby potentially improving resilient performance through the work of sense-making and interpretation, risk management, and maintaining morale. This has implications for the RBV theory, as it may assist health service delivery providers in cultivating distinctive competencies (via sense-making and interpretation), managing resources efficiently to mitigate risks (through risk work), and optimising human resource potential (through work on morale).

The use of Artificial Intelligence has been hailed by political leaders to offer a solution to NHS pressures and staff shortages [20]. Our work implies these meetings and the people who take part in them are not fully replaceable by alternative methods. Some of the information brought in is numeric and could potentially be gathered and interpreted using Artificial Intelligence, but some of the information related to unmeasurable one-off events or the “emotional state” of departments can only be captured and made sense of by humans.

In the discussion, the authors might want to provide more direct suggestions or recommendations for government and highlight lessons for other health facilities. Additionally, the article will benefit from including more clinical implications of the findings. Further discussion and literature review would also be appropriate to compare and contrast similar forms of operations meeting in other regions or countries as well as the respective effects on the health system. This would ensure a comprehensive and more thorough review of the subject.

Moving forward, greater understanding of the form and function of the work conducted in these meetings is vital to be able to compare between different hospitals, healthcare systems and with other industrial sectors. Internationally operations management is diverse with differences in approach and outcomes between low and high income countries [21], and our work was aimed at understanding function of the meeting rather than approach to standardising form. However regardless of the size of the operations team and its objectives we believe it is likely the three primary activities will have face validity in a variety of settings. Considering the lessons learnt about improving patient safety from other sectors (especially aviation), we may also learn lessons to improve Operations meetings. Finally our findings have identified the diverse range of staff attending these meetings (which is not limited to purely managerial staff) and may therefore highlight multiple training needs for participating in and running meetings.

### Strengths and limitations

This qualitative study used independent observations of Operations meetings and interview data across three hospitals, conducted by non-clinical researchers with contribution from clinicians including individuals with operations management knowledge and expertise. Although we selected three hospitals with variability in terms of locality, the types of data systems used, and the format of Operations meetings (in-person and virtual), our findings may not represent the full range of approaches to Operations meetings in NHS Trusts across the country, particularly in smaller hospitals.

Interviews were audio-recorded (with consent), but we did not audio-record Operational meetings due to ethical issues around sensitive data including confidential patient information and hospital operations; so details may be missing from the anonymised notes taken by observers.

Although this research setting enabled us to capture and understand resilience work that occurred during Operations meetings, further studies could explicitly explore the barriers and facilitators of successful outcomes of these meetings.

## Conclusion

Our study demonstrates how work undertaken by staff in Operations meetings functioned to achieve resilience in service delivery in routine care on a day-to-day basis. Decision-making around capacity and demand in these three trusts was not purely a matter of dissembling hard centralised data, but rather involved a shared and distributed understanding of the unfolding situation on a daily basis informed by local intelligence, experience and ‘a feel for the game’. We identified three types of work: Sense-making and Interpretation, Risk work (which contributed to maintaining organisational function) and Maintaining morale (which supported individual staff resilience). This framework, if validated, could be used to compare and contrast hospital Operations management approaches to enable determination of best practice.

## Supplementary Information

Supplementary Material 1.

Supplementary Material 2.

Supplementary Material 3.

## Data Availability

The datasets used during the current study are available from the corresponding author on reasonable request.

## Abbreviations

NHS: National Health Service
CARE: Concepts for Applying Resilience
