# Supplementary material for: How do staff work in NHS hospital operations management meetings to support resilience in everyday service delivery? A qualitative study
Source: BMC Health Serv Res. 2025 Jan 21;25:113. doi: 10.1186/s12913-025-12229-3 (PMC11749087; doi:10.1186/s12913-025-12229-3)
Supplement: Supplementary file 2 — Supplementary Material 2. [file 12913_2025_12229_MOESM2_ESM.docx]

Appendix One

How do staff work in NHS trust Operations Management to support resilience in everyday service delivery? A qualitative study

NOTE ANY ACRONYMS

Orientation & conduct

How does the lead define and orient participants to the purpose of the meeting? Are goals/expectations explicit?

How does this meeting link to and build from the previous meeting? Do they check that actions from previous meetings have been taken?

Describe the way the meeting is conducted / the structure and routine of the meeting.

Are there assumptions about shared understanding? Any surprises/disruptions – how do these play out?

Participants

Number of participants; do people arrive at the beginning and stay, or drop in and out? Any discussions about this?

Do people introduce themselves / make clear which area they are representing?

Information exchange

- What types of statements or questions are made by the ‘silver’ lead to seek the status of each directorate/division, or find out what help needed?
- What information is discussed? And how is it used?
  - *hard data* (bed occupancy numbers, staffing levels and sickness, outliers, forecast data – people in ambulances, due to be transferred, due for discharge)
  - *soft information* – e.g. very sick patients, difficult family, end of life, ‘feel of the ward’ e.g. frantic,calm
  - problems/complaints…
- What are the *timeframes* of information shared and discussed (e.g. today, this evening, tomorrow…)
- *Describe the discussions around the information shared in the meeting,*
- Any examples of when data/information is needed, but is not available? What happens?

### Decision-making

- Note anything about how people *make the case* for their division (terminology e.g. ‘desperate’, ‘tricky’) and *negotiate* their position (e.g. exceptional circumstances); and *offers of help* from other participants. Are there any conflicts/tensions expressed and how are they managed?
- Is anything mentioned as a particular overall priority (e.g. making space for elective work, for ambulance arrivals…)
- *Describe decisions*
  - *What types of decisions are being made and timeframes of those decisions/actions e.g. short term staffing fix; ‘keeping an eye on things’; reporting back; longer term planning (i.e. beyond the next ops management meeting).*
  - *What do decisions involve e.g. suggestions of solutions to problems (e.g. ‘get portering…’); use of staffing beds and and space; addressing patient flow e.g. faster discharge…*
- Who makes the decisions, and how (e.g. consult then make autocratic decision; discussion and consensus; delegated…). Is it clear who has made the decision?
- Do people seem satisfied with decisions/is there any sign of dissatisfaction?
- *Is it clear what decision has been made and what actions need to be taken as a result of the meeting? And is it clear who is responsible for carrying out the decisions?*
- Do they discuss anything that constrains the decisions that can be made in the meeting?

Ending the meeting

- How does the meeting end?
- Is there any planning for the next meeting, requests to report back at next meeting?
