# Supplementary material for: How do staff work in NHS hospital operations management meetings to support resilience in everyday service delivery? A qualitative study
Source: BMC Health Serv Res. 2025 Jan 21;25:113. doi: 10.1186/s12913-025-12229-3 (PMC11749087; doi:10.1186/s12913-025-12229-3)
Supplement: Supplementary file 3 — Supplementary Material 3. [file 12913_2025_12229_MOESM3_ESM.docx]

Appendix 2 – Supportive Quotes and Analysis

## Sense-making and interpretation

A key function of operations management meetings was to enable operations managers and divisional leads to engage in sense-making and interpretation to construct a shared understanding of the current state of the trust. The type of information or knowledge used to gain a shared representation of the situation included centralised bed-state data, ward-level information about current bed states and staffing, soft intelligence about local conditions, and discharge predictions.

### Bed data

Centralised data held on bed-state data systems included bed state numbers, number of critical care beds, step down beds, numbers of patients waiting, number of ambulance calls, number of infections, number of beds closed and staffing numbers. Operational leads also had information about the organisation’s Operational Pressures Escalation Levels (OPEL) status (a standardised measure of pressure on the organisation based on assessments of capacity and demand numbers across the organisation, reported as a number on a scale from 1 to 4, where 1 corresponds to meeting anticipated demand within available resources, whereas 4 indicates major pressure that is compromising the trust’s ability to provide comprehensive, safe care).

Key elements of this information were shared by the chair to provide a high-level overview of the state of the organisation.

*We remain in OPEL 3. ED, surgery and clinical support are in RED^[[1]](#footnote-1)^ status, Medicine and CAS in critical status. A&E opened its first corridor, they have some planned transfers but no beds. 210 attendance to ED – expecting 569 and still 134 patients in the unit. Length of stay around about 24 hours, waiting for beds to be declared. (Organisation B, OBS01B).*

This data provided the foundation for flow management and planning. However, centralised data had limitations. It was not always complete, and bed states and staffing numbers in particular were not always up to date and could change rapidly.

*So yeah, so I’ve had it, I’ve had it both ways, I’ve had it where the data’s been accurate, and I’ve been there, I’ve had it when I’ve walked onto a ward and there’s empty beds and I’m like well what’s, you know, can you, can you clear them off the, off the, off the system. […] Or can we, can we update the system? (Organisation A , site 03)*

Divisional staff reports during the meeting provided real time updates to the formally recorded data, summarising their capacity and highlighting any issues.

*Women’s: Gave number of empty beds “9 with 2 later. GAU at (name of hospital) have 1 now, 3 later.” [Meeting participants] talked about ward 31 to look to transfer if busy. “Neonates at Opel 3 = 37 babies at [name of hospital]. Obstetrics – acuity 65” (Organisation A , OBS 03).*

Having a clear awareness of the overall state of the organisation was supported by review of the data held on the central system, but also involved a shared and distributed understanding of the unfolding situation on a daily basis, and the local conditions that could impact on this. Bed state information had to be contextualised through sharing and taking into account local intelligences from across the divisions.

### Accessing soft intelligence

Locally-held soft intelligence included: knowledge about problems or delays arising from estates and facilities issues such as lift breakdowns of cleaning requirements; knowledge about patients who might be challenging, or require additional support or staffing resource, and/or equipment such as specialist beds and hoists; staffing changes and sickness; and informal knowledge and experience of their division and site.

*Issue with a lift on [name of ward] re patients meals and discharge and put update in chat. […] Need to get that sorted. Facilities person said he was having a meeting with estates in 20 mins (Organisation B, OBS05).*

*The surgical female patient needs a side room. Lead said it would be a struggle for oncology – surgery could accommodate. (Organisation A , OBS 02)*

*Staffing mentioned also. Locum doctor for the afternoon so only one down in the afternoon. Nursing staffing now 3 down rather than 5 down. Covered the GP gap. (Organisation C, OBS 01B)*

*Got 2 spaces closed in majors – waiting for some type of cleaning. (Organisation A , OBS 04).*

Chairs described the importance of accessing soft intelligence from divisional leads about the state of their bed capacity and staffing

*That’s the bit that you’ll pick up, your soft intelligence, is the bit when you’re actually having that conversation with people, they’ll tell you, you know, […] It looks like that but actually it’s not that, […] or we, you know, staffing issues, you can’t see the staffing issues from, from that screen, it doesn’t tell you, so you have to use all the forms of communication with them to actually ask if we’ve got any staffing issues and can we use those* (Organisation B, INT05).

### Anticipating future states

The shared understanding constructed within operations management meetings also needed to encompass possible future states. They had to consider events or conditions that could place additional demand on the service over the next few hours such as festivals, industrial action and potential ambulance transfer from another Trust.

*[Ambulance Service representative] good position for us this afternoon, 12 waiting calls, of those 1’s got PRU on it and 4 accepted by CAT. 39 active calls. Staffing not good overnight -3 shortfalls. Neighbouring Trust A&E has a divert in place to another neighbouring hospital and [has] refused another one. A hospital in a neighbouring county is busy. Currently 13 P s] O[n]A[mbulances] at Neighbouring Trust A&E and another 3 inbound and 5 awaiting handover. Chair: did you see much activity with strategic conveyance coming our way? Response – nothing at all. Chair: if they do request help, they need to follow the right channels. We’ll keep an eye out. It’s getting a little bit tighter. (Organisation A , OBS07)*

Those in the meeting also needed to consider planning for discharges within their wards and divisions, and assess whether they would have enough beds available if there was increased demand in any other part of the organisation and whether they had the staff to meet changing demand. In Organisation B, predictive information on potential discharges was available on their centralised systems, whereas in Organisation C and Organisation A this tended to be shared as informal knowledge by representatives from each division.

*Discharge news 78 simple and 3 complex patients discharged. There were 32 on the complex list and 29 have gone. 5 patients waiting for community hospitals. (Organisation A , OBS 08)*

Where information about predicted discharges was relayed in the meeting, there was some doubt about the extent to which predictions could be made accurately. Such data was specific, partial and constructed and required more work to interpret.

*A bed is not empty…Until there’s not a patient in it* (Organisation C, INT 005).

*We have a number on our reports that predicts how many people are going to come into the hospital […] and we have a very out of date, very out of date, predict[ion of] how many discharges we’re going to have*. (Organisation B, INT 06)

### Discharge Predictions

Data and intelligence shared had to be interpreted to construct meaning and identify implications. Chairs had to make sense of the information to understand the implications for capacity and flow across the organisation, in order to identify areas of risk and prioritise actions. Divisional representatives had to listen, translate and make sense of each piece of information shared and undertake work to understand what it meant to their division and how it might impact on other parts of the organisation. Chairs and divisional leads discussed how data from one division impacted on demand and requirements from other divisions in order to manage flow. Participants had to re-assess their own division’s capacity following the breakdown of the types of patients they were receiving, who they could move from where and whether they could take additional patients on specific wards.

*The chair asked about <organisation 3> and the numbers of beds were shared. […] The chair commented that this was really good capacity, however, there are a lot of patients in A&E with no plans and there are 30 patients going on the bed list in a few hours and that will “be your [full] capacity and more”*. (Organisation A OBS09).

*Surgery [representative]: 12 patients on virtual ward, with 4 today so looking better. Chair asked for a breakdown 1 vascular 2 neuro, 1 general surgery (I suppose). Issue with gastro and neuro and have placed all electives. Heavy day. Have reviewed x ward and 2 patients are going. [They have] given 2 beds to medicine and number of beds to cardiology (not sure how many). Treatment centre to confirm capacity and move some patients. Discharge not looking good. We can go 1 over on y and z wards. (Organisation B, OBS 04)*

Chairs in Organisation A in particular, described how they worked to make sense of the data in terms of implications for demand, capacity and flow planning going forward. They described the critical role of informal, intuitive knowledge and experience in enabling them work out, in collaboration with divisional leads, where pressure points were likely to be, and potential risks. This type of knowledge remained unquantifiable and unmeasurable.

*But you know when you’ve been doing it for a while […] you have a background in your head of what… So […] Surgery might say they’re minus 23, but that their figures will assume that they’ve got 23 people in triage, and actually experience tells you that at least 50 percent of those people will go home. So you look at that and think well that might be OK. I mean in theory you could have one of those days where every single patient comes in, but generally they don’t. So you’re thinking if they’ve got 10 beds and 20 people in triage, they’re probably gonna get to a zero, and you sort of balance the risk off in your head. But experience tells you that. (Organisation A , INT01)*

*So there’s a lot of stuff in your head that translates the information into something that, that makes sense, and how you would automate that on any sort of data pack I’m not sure. So you could have a piece of data from [central bed-state system] that tells you what the empty beds are, but actually we need to know more than that, we need to know what’s empty plus who they know is going home today, and then the queries will be the ones where they’re waiting for a blood test or something like that, or a surgical review, and they might, they’re probably gonna go home later. (Organisation A , INT01)*

In Organisation B, by contrast, they appeared to rely heavily on the data to make decisions but also required information about the where staffing skills gaps might lie, as this interviewee points out:

*I think we are quite data heavy. I mean I think, I suppose you would… You know, there’s other stuff going on, so like if we know we’re going into strikes, or you know, if there’s a particular, you know, there’s a big event, quite often we have festivals and stuff like that at the weekends, then you know, there might be some different decision making around that kind of stuff…..But I think a lot of our stuff is quite data heavy, but then also I suppose stuff around staffing as well which is not necessarily a defined number, it’s kind of, you know, sometimes it’s the, not the quality of staff but the skill mix, isn’t it? (03 Organisation B).*

Sense-making and interpretation of data and intelligence took effort and skill. Many interviewees reported that they had chaired the tactical meetings for a considerable time. None of them had received any formal training to perform that role and all had learnt from others whilst ‘on the job’.

*So there is no formal, you know you can go and shadow somebody, but, but there is no kind of, you know, you need to do, you know, here’s kind of a checklist and you need to have done this, or you need to have done the so many shadowing shifts, there’s none, there’s none of that. (Organisation A , INT 3)*

## Riskwork

Riskwork in this context describes the human effort, in combination with the material infrastructure of data systems and operations management meeting routines, that come together to understand and organise risks.

### Identifying risks

Area of risk could be revealed through the sharing and interpretation of information about bed states, capacity, anticipated future states and local conditions. They could also be brought to the fore through deliberate highlighting of known risk areas by the chair or divisional representatives.

*ED representative […] wanted to flag that ED was challenged from a nursing perspective. Seven gaps which means that they can only take four ambulance handovers. That may be increased to eight but there is a risk to ambulance handovers (Organisation B, 03A)*.

*The hospital was under pressure, many people were waiting to be seen which was highlighted as a huge risk for them* *(Organisation C, OBS02A).*

### Prioritising risks

After identifying potential risks, the chair set the priorities in discussion with participants articulating what staff needed to focus on to improve the situation, or ensure that it did not deteriorate further. This informed the chairs review and discussion of the options available to them, action planning and task allocation.

*Close ED corridor by midday at the latest. Involves reducing the wait for B3 – hopefully by the next meeting. Delayed step downs from the 29th need to be placed by the 1pm meeting. Need to fill all available capacity within division. (Organisation B, 03A)*

*The focus was on making sure that people who are medically fit and waiting to be discharged are actually discharged and not sat around waiting on a ward and taking up an important bed space (Organisation C, OBS02A).*

### Allocating responsibility

After highlighting areas to prioritise, the chair and participants at the meeting aired and discussed potential options to mitigate identified risks. Divisional leads reported particular issues or problems their division was facing. This usually involved complex patients or IT or equipment failures.

*Medicine - very critical, already boarded. Very reliant on discharges. Discussion about a speciality patient on B3 whose wife can’t take him home. Lead asked ‘why wasn’t this gentleman sent to an outlying ward? Not sure will find out (Organisation B, OBS 07)*

Based on collective understanding obtained in the meeting; staff had to find solutions to the situation. Their options were often limited but they were aired and negotiated. These focused, for example, on whether to close or open the discharge lounge, or move patients from one place to another.

*Discussion about if we need to close the discharge lounge can we use the nurses better elsewhere? Discussion about night staff. Chair said will look to see what’s happening in the discharge lounge later, carry on for now. If we are to close discharge lounge will then relocate nurses, said let me make some phone calls and will let you know. (Organisation A , OBS 03)*

*ED has a stroke patient with Covid. Mentioned that to place this patient on the stroke ward they would need to move 3 outliers and that a bay would need to be flipped. A side room would need to be found and she said that that would be problematic. She has asked the message to be passed to someone (didn’t catch who). (Organisation C, OBS 01B)*

Different options were available across the different trusts.

*Organisation C ‘had a full capacity protocol in place which enabled them to make decisions around where they could reverse board patients based on how many patients were in the department, how many decisions to admit they had broken down by speciality, what discharges were confirmed and what queries they had’ (Organisation C INT 002).*

Chairs had to make sense of which staff were required in order to open beds. It was only through relaying information about skill mix that they could understand how best to proceed.

*Chair: ‘Can you open another 4 beds? Nursing manager – staffing: No. Skill mix of nursing in children’s. They need to be experienced and can’t support. There is a ITU nurse that can support them in ITU.* *(Organisation A , OBS03).*

Divisional leads reported when they had no capacity or had staff shortages then all options needed to be considered to resolved the situation

*Oncology and haematology on a take of 7 overall -6 . Oncology and haemo challenged. Not been able to take our patient out of ED. Currently working on an outlier list. Been escalated to CD and deputy CD who are trying to work through some plans to get some flow. Something about a patient on ward 39 to give us a bit of movement. (Organisation A , OBS 06)*

*Medicine asked [the lead] you sent me a nurse but they finish at 1pm. Can you send me some nurses to muck in? Yes. Thank you (Organisation B, OBS08)*

In the following extract the chair also identifies the option of using the Cancer division’s spare capacity for other patients.

*Cancer were at amber. Have capacity. Need to place surgical and medical electives. No capacity on oncology. Bed manager is working on getting an oncology bed. Lead – great something about using capacity for outlying respiratory patients or those on HCOP but to check with bronze. (Organisation B, OBS07).*

However, there were instances where the options aired were not viable. In the following extract other options were not discussed following a request for others to help transport patients.

*Chair asked if a specific team could “crew up” to help. ED representative who was reporting said that she thought this might be possible but also gave some reasons why this might not work. She said that the problem is that they support another area now which means that they are very limited on what they can do. (Organisation A , OBS 01)*

Action-planning & goal setting (including following up problems) was primarily undertaken by the chair who summed up actions at the end of the meeting by asking staff to focus on key areas and prioritise certain issues. We found that there were instances of actions being specifically targeted at participants in order to resolve an outstanding issue.

[*The chair] established which patient they were talking about and then asked [Duty manager of hospital] to take this up as an action and to link with [senior sister] to sort it out (Organisation A , OBS 01)*

*Chair [asking facilities person] - can we get a timescale for fix on [ward lift repair]. Facilites – will chase. Lead Let me know if you need contractors pushing as it’s our highest discharge area. (Organisation B, OBS 06)*

However, there were also instances where chairs summarised actions at the end of the meeting but often without giving a clear timeline, or whose responsibility it was to undertake each task. Moreover, we found few examples given of how these goals were to be achieved.

*Chair summed up with their priorities which were to support E12, start theatres, work through B3 list and look at everything on discharge – 80 queries so push on transfer of care and discharge. Get D2As submitted*. (Organisation B OBS05)

*Actions starting today – main target is for patients not to breach the 4 hour / 12 hour ED targets. Zero tolerance of patients waiting 12 hours in ED and that actions need to be taken before this happened* *She concluded her bit by saying that that numbers were very high and that they ( the division leads) had to submit plans throughout all of the divisions to submit discharges. She asked them to use the discharge lounge to create flow.* (Organisation C OBS01).

*More of what they have been doing in the morning. Keep first contact trolley free so that can off load the ambulances. RESUS with a couple of beds so can turnaround any urgent patients. Keep the corridor closed. The opportunity is there because discharge profile is better but aiming for 300, it needs to be. Need the D2As forms to iDT this afternoon. (Organisation B OBS 02B)*

Often goals were vague and included such phrases as “keeping an eye on”, “staying on top of” or “push for discharge”.

“*I need everybody’s best efforts to get their patients out”. Need to get patients in the right place today*. *Chair then said can everyone just nudge the Drs to get as many TTOs done as they can. We need to prepare ourselves for tomorrow.* (Organisation A , OBS 06)

*Stay on top of the D2A process – to do this by chasing the divisional bronzes and chase down the D2A forms. Want them all to be in the hub and the hub to be overwhelmed. So patients can move out.* (Organisation B OBS02A)

When there were senior people at the meeting, they were often able to confirm the way forward during the meeting itself rather than later. For example, there was an issue with a CT scanner at one hospital which meant having to transfer patients to another hospital in the vicinity. Having an operations manager at the meeting meant that the decision to get transport arranged for patients was taken immediately.

*The chair clarified that they do not have a functioning CT scanner at [name of hospital] When this was confirmed, the chair asked an operations manager to join in the conversation. This person said the situation was “slightly concerning” – said that she would set aside an ambulance dedicated to do those transfers (Organisation B OBS 01A).*

## Supporting Resilience

### Recognising success and displaying empathy

The work of the operations management team involves having to manage a high level of demand with limited capacity on an ongoing, day to day basis. They had to manage and balance risks, negotiate and make difficult decisions, constantly revisiting the situation with frequently shifting resources. The interviewees reported that they faced chronic stressors every day and that each day felt the same as the last. It could be draining and demoralising for all involved.

*The meeting is a bit Groundhog Day because you know, there's long waits on ED, there's long. There's a lot of patients waiting for medicine in ED, so it will be the same. You'll hear the same thing every … you know, I'm not exaggerating much by saying every day you'll hear the same problem, so that can be quite tiring or jading is the right word…We're always, always under pressure. Certainly, in medicine always under pressure to provide more beds and every day is a pretty bad day [laughs] to be honest*. (Organisation A INT01A).

*A bit like it’s Groundhog Day, if I’m honest. But I think that’s probably indicative of the situation we find ourselves in at [name of Trust] on a regular basis. We are regularly in OPEL 4, so a heightened status of escalation. We very regularly come into an overcrowded emergency department with significant patient waits. So it feels really hard, if I’m honest. It feels really, really difficult to come in and do that again and again.* (Organisation B, INT02).

*I do always feel it’s a bit like Groundhog Day. Because there are often not enough beds and there are often limited numbers of solutions*. (Organisation B, INT06)

Very often, this meant that participants were wary and anxious about what they were about to face when they accessed the data before the meeting.

*You just think oh my goodness, what am I coming into? More than anything, rather than it being…You just have that heartsink as soon as you turn it on, going what am I coming into? (INT 04, Organisation B)*

In many meetings we observed work that primarily functioned to support and reinforce resilience. Chairs worked to ensure a sense of community and belonging, with a sense of collective working towards common goals. Given the sense of futility that could be attendant to operations management meetings, work to establish clarity of purpose and achievable goals played an important role. In some cases chairs worked to keep the focus on issues they could all address, rather than those that they could not. With the emphasis on targeted discharges on a daily basis at Organisation B, it became possible to observe and measure this particular aspect of success.

*Lead summed up with their priorities which were to support [department], start theatres, work through [list] and look at everything on discharge – 80 queries [possible discharges] so push on transfer of care and discharge. (Organisation B OBS05)*

Chairs also emphasised the maintenance of momentum in addressing patient flow as a goal in itself, even if the absolute overall position of the trust in terms of OPEL status changed little from meeting to meeting.

*His emphasis was on movement and that they needed to keep pushing to keep things moving “We just need traction on that movement”. This drive to create movement is something that was emphasised many times during the meeting. “Let’s keep the ball rolling”.*  (Organisation A OBS01)

Displays of resilience and coping from divisional leads also seemed important for collective morale. The participants let the chair and others know how their division was doing, whether they were coping with demand or whether they needed support. When faced with problems to solve, divisional leads often responded by stating that “we will do our best” or that they could cope. As many interviewees reported, none of those attending the meeting wanted to do a ‘bad job’. They were all there to try and do their best, along with their support teams to tackle the issues they faced. We observed work undertaken by participants to demonstrate that their own division was up to the task, and that they could cope with the pressure.

*Also mentioned two members of staff that had had to go home one due to being on an early and the other due to sickness. But she felt that they could manage with what they had (Organisation A OBS01).*

*Working through outlier capacity but nothing to worry about at the moment. Said that they will be fine and “will hold their own this afternoon” (Organisation B, OBS 01B)*

*Chair asked ‘are you comfortable to keep the flow - it’s a lot in respiratory? They said they could manage the backstream. Chair said that it was an amazing turnaround massive shout out. (Organisation A , OBS 03)*

We observed chairs working to maintain morale through encouragement, praise and gratitude.

*First mentioned was the success in getting the corridor shut, she thanked everyone for their support and that ticking that box meant it was a better experience for the patient even if it made ED busy.* (Organisation B, OBS02B)

*She ended by saying that she would see them at 5pm and congratulating them as they had done everything that they had tasked them to do. Encouraged them to “keep their foot on the gas” so that they were in the best possible place at the weekend. (Organisation B OBS 02B)*

*Great team work in getting patients in right place. Keep going*. (*Organisation A , OBS 05*)

It was therefore notable when emotional support was not forthcoming, as evident in the following example.

*Person 07 arrived and plonked her papers on the desk. “I don’t think they will be happy with me, not hit my target although we do have 10 less patients than before”. Person 08 did say and not in a joking way, that is not many in three hours. (Organisation C, OBS 02B)*

There was also considerable emotional work done by the chair through signposting divisions to sources of support. There were more instances of emotional work conducted in Organisation A than in the other sites, despite more observations being carried out in Organisation B.

*Chair reminded them to ask the home birthing team for support if they needed it. <PERSON 3> agreed that they were their “go to” people for support. (Organisation A , OBS 01)*

*[Possibility of] ward 31 transferring beds over to ORGANISATION 4 maternity. Lead: If that happens will you make sure that a message goes out on the [name of hospital] ops so that everyone knows. Anything that worries you? Staffing ok at ORGANISATION 2, not the best at ORGANISATION 4 and that they’ve been in touch with the home birth team and making the staffing person aware (on call). Staffing person – the Matron had no concerns at the 4.30 meeting. Lead: Ok thank you make sure they are aware they can call us (Organisation A OBS 07).*

1. Measurement of criticality: Red, Amber, Green. Status red = no beds [↑](#footnote-ref-1)
